# Identification and characterization of intracerebral hemorrhage events in elderly veterans with alzheimer’s disease in the veterans affairs healthcare system

**DOI:** 10.1038/s41598-026-58486-y

**Published:** 2026-06-19

**Authors:** Peter J. Morin, Vanesa C. Andreu Arasa, Ying Wang, Joel Reisman, Dan Berlowitz, Wen Hu, Raymond Zhang, Babak Haji, Ran Gao, Amir Abbas Tahami Monfared, Quanwu Zhang, Weiming Xia

**Affiliations:** 1 Department of Neurology, Boston University Chobanian and Avedisian School of Medicine, Boston, MA USA; 2 Department of Radiology, Boston University Chobanian and Avedisian School of Medicine, Boston, MA USA; 3 Research Service, VA Bedford Healthcare System, Bedford, MA USA; 4 Wentworth Institute of Technology, Boston, MA USA; 5 Department of Biological Science, Kennedy College of Sciences, University of Massachusetts Lowell, Lowell, MA USA; 6 Department of Public Health, Zuckerberg College of Health Sciences, University of Massachusetts, Lowell, USA; 7 Clinical Evidence Generation, Deep Human Biology Learning, Eisai Inc, Nutley, NJ USA; 8 Epidemiology, Biostatistics and Occupational Health, McGill University, Montreal, QC Canada; 9 Department of Pharmacology, Physiology and Biophysics, Boston University Chobanian & Avedisian School of Medicine, Boston, MA USA; 10 Geriatric Research Education and Clinical Center, Bedford VA Healthcare System, 200 Springs Road, Bedford, MA USA

**Keywords:** Alzheimer’s disease, Cerebral amyloid angiopathy, Hypertension, Intracerebral hemorrhage, Mild cognitive impairment, Diseases, Health care, Medical research, Neurology, Neuroscience

## Abstract

Cerebral amyloid angiopathy (CAA) and hypertensive (HTN) small vessel disease are causes of spontaneous intracerebral hemorrhage (ICH). We identified ICH rates in patients with all-cause mild cognitive impairment (MCI) or Alzheimer’s disease (AD), and explored feasibility of using location-based approach to differentiate CAA- and HTN-related ICH using comorbidities in electronic health records (EHRs). This administrative study combined Veterans Affairs Healthcare System plus Centers for Medicare and Medicaid Services (VAHS/CMS) databases. Patients with MCI/AD aged ≥ 50 years (2016–2023) were 1:1 matched to non-MCI/AD controls. Inpatient primary discharge International Classification of Diseases-10th Edition (ICD-10) codes identified acute ICH; anatomical location within codes classified events as likely CAA- or HTN-related ICH. Incidence rates of ICH after MCI/AD were summarized. Cluster analysis of variables related to ICH was used to describe whether data-driven groupings matched clinician-postulated classifications. The MCI/AD cohort (*n* = 747,475) and controls were aged 77.7 ± 10.1 years (96% men, 75–76% White, 87–88% non-Hispanic). Demographic- and comorbidity-adjusted rates of ICH/1000 person-years were 0.84 (overall), 1.05 (MCI/AD), and 0.68 (non-MCI/AD). Adjusted events/1000 person-years were higher in MCI/AD vs. non-MCI/AD cohorts: CAA-related ICH, 0.19 vs. 0.12 (Incidence Rate Ratio (IRR) 1.63; *P* < 0.001); HTN-related ICH, 0.16 vs. 0.12 (IRR 1.35; *P* < 0.001); non-specific-ICH, 0.68 vs. 0.43 (IRR 1.59; *P* < 0.001). Hierarchical clustering analysis of our cohorts revealed an association of CAA-related ICH with older age, cardiovascular and rheumatic disorders, and an association of HTN-related ICH with cerebrovascular disease, hypertension and diabetes. In sum, the estimated incidence of ICH over the study period was 0.84/1000 person-years. CAA-related ICH incidence in MCI/AD was 63% greater than that in controls. Outcomes from cluster analysis are consistent with ICD-based CAA- vs. HTN-related ICH classifications. These findings support future exploration of using ICD coding-based ICH event identification in EHRs and claims databases for epidemiological studies.

**Supplementary Information:**

The online version contains supplementary material available at https://doi.org/10.1038/s41598-026-58486-y.

## Introduction

Cerebral amyloid angiopathy (CAA) and hypertensive small vessel disease are major underlying causes of spontaneous intracerebral hemorrhage (ICH)^1,2^. Alzheimer’s disease (AD) is associated with a higher prevalence of CAA than the general population^3,4^ and has an increased risk of hemorrhagic stroke^5^. Amyloid deposits in vascular smooth muscle layer may weaken blood vessels over years and lead to CAA-related hemorrhages and have a high re-occurrence risk^2,4,6,7^. In cases of hypertension (HTN)-related ICH, the major underlying cause is chronic vascular stress with intimal proliferation, with associated hemorrhages typically occurring in deeper brain structures such as the basal ganglia, thalamus, brainstem, and cerebellum^8^.

Cerebral microbleeds (CMB) are an established risk factor for spontaneous ICH^9^. Increased microbleed burden has been associated with higher risk of ICH recurrence especially in those with hypertension and diabetes, where CMBs mainly occur in deep brain regions (e.g., basal ganglia and infratentorial regions)^10^; in contrast, CMBs located in superficial or lobar regions of brain are pathologically characterized by the gradual deposition of amyloid β protein (Aβ) in vessel walls, i.e., CAA, which are common in patients with mild cognitive impairment (MCI) and AD^11,12^. Lobar microbleeds, particularly, have been reported to increase risk of incident stroke in patients with AD^13^. CMBs are a key imaging marker of CAA and small vessel disease, and the location of CMBs may serve as a surrogate to associate CAA- or HTN-related ICH.

Several studies have validated the use of diagnostic coding to identify acute ICH and hemorrhagic stroke events^14–16^, including the identification of acute ICH in patients with all-cause MCI or AD^17^. We hypothesized that ICD-coded ICH events could be empirically differentiated into CAA- and HTN-related clusters based on anatomical location and comorbidity patterns in real-world data.

This study aims to identify overall and subtype-classified rates of ICH in patients with MCI and AD, and to explore the feasibility of associating CAA- and HTN-related ICH with comorbidities from the electronic health record (EHR).

## Methods

### Data source and extraction

This was a retrospective analysis of the Veterans Affairs Health System (VAHS) VA Informatics and Computing Infrastructure (VINCI) database^18^ combined with the VA Information Resource Center (VIReC) Centers for Medicare and Medicaid Services (CMS) research database, allowing capture of Veterans’ use of both VAHS and CMS services^19^. All Veterans aged 50 years and older were extracted from the VAHS VINCI database from January 2016 through December 2023. Additionally, pertinent data corresponding to these Veterans available in the VIReC CMS database were extracted through December 2023.

The VA Bedford Healthcare System Institutional Review Board approved this study, and data were all fully de-identified before access.

### Study population/case definitions

The overall study population consisted of Veterans with MCI/AD (cases) and a matched cohort of Veterans without MCI/AD at any point (controls). For the case cohort, Veterans aged 50 years and older who had all-cause MCI or AD with earliest date 2016–2023 were identified from a database that combined VAHS-VINCI and VIReC-CMS databases (herein referred to as VA/CMS) for encounters in October 2009 through December 2023. In order to reflect the natural history of ICH, the study cohort was identified largely before patients were prescribed anti-amyloid therapy (AAT) in the VAHS. Few Veterans were prescribed AAT in 2023, excluding the subjects with AAT-induced microhemorrhage in our cohort. *International Classification of Diseases (ICD)- 9-Clinical Modification (CM)* or *ICD-10-CM* diagnostic codes for MCI (*ICD-9-CM* 331.83; *ICD-10-CM* G31.84) or AD (*ICD-9-CM* 331.0; *ICD-10-CM* G30.X) were identified from the EHR. Patients were included in the case cohort if they had 1 qualifying diagnostic code or had 2 qualifying clinical notes that were separated by 30–364 days. Our clinical notes-based key word identification approach has been described previously^20^. Whether a patient was defined as MCI or AD in the analysis was based on whichever diagnosis met the case definition first; date of first code/note was defined as the index date. The cohort of Veterans with MCI or AD was matched 1:1 on age by year and sex without replacement to Veterans with no coding for MCI/AD (non-MCI/AD control cohort). Controls were further matched to cases, if there was a clinical encounter occurring during the year of the case’s index date; this encounter date was defined as the control’s index date.

ICH was defined based on ICD-10 coding, previously validated using hospital discharge notes review^17^. The validation yielded positive predictive values of 72–81%^17^. Briefly, inpatient nontraumatic ICH events were identified (2016–2023) using select ICD-10 codes (Supplemental Fig. 1). In order to identify ICH events, hospital discharge data files were used and ICH diagnostic positions were determined^13^. Due to variations in coding processes and nomenclature between the VAHS and CMS structured databases, ICH event identification was customized: in the VAHS, both the principal discharge diagnosis (i.e., the code that contributed most to the length of stay) and ICD-10 codes listed in the first position in the discharge diagnosis list were used; whereas for CMS, ICD-10 codes in positions 1 and 2 of inpatient claims were used. Primary ICH was limited to VAHS principal discharge diagnosis and position 1 on CMS claims. ICH events were classified as related to cerebral amyloid angiopathy (CAA-related ICH) or hypertension (HTN-related ICH) using a system (shown in Supplemental Fig. 1) developed collaboratively by a neurologist and neuroradiologist who postulated the classification by relying on information regarding anatomical location available within ICD-10-CM code descriptions^17^. This clinician-postulated system designated whether the location was more likely to indicate a CAA-related ICH or HTN-related ICH event based on collective clinical experience and scientific publications^10,21–23^. If ICD-10 codes did not provide adequate brain subregion/location-related information to permit classification as likely CAA-related ICH or HTN-related ICH, then events were classified as non-specific ICH. The ICD-10 codes for non-traumatic subarachnoid hemorrhage (SAH, I60) were excluded. Use of the term “ICH event(s)” in this study encompasses all ICD-10 codes listed in Supplemental Fig. 1.

A sensitivity analysis of incidence rates was performed in which I61.0 was classified as CAA-related ICH, and I60.8 and I60.9 were included as CAA-related ICH to assess the impact of incorporating SAH related diagnostic codes. Of note, nontraumatic SAH ICD-10 codes (I60.8, I60.9) were included in the sensitivity analysis since these codes have been used to^1^ capture CAA-type events in clinical practice and^2^ research data suggests an association of SAH with CAA and intracerebral hematoma^22^. The sensitivity analysis was based on inpatient claims in ICH diagnostic positions 1 and 2.

### Statistical analysis

Baseline characteristics of the study population, including demographics and comorbidities were summarized. Comorbidity covariates included Charlson comorbidity index components, clinician-determined ICH-risk factors, and other co-morbidities common in AD (Supplemental Table 1)^24^. First, we sought to estimate the overall and subtype-specific incidence rates of ICH among the study sample. Incidence rates of ICH (per 1000 person-years, for CAA-related ICH, HTN-related ICH, and non-specific ICH events) were estimated using multivariable Poisson regression models with a log link and an offset for person-time, adjusted for demographic variables and baseline comorbidities. Incident ICH was defined as the first recorded ICH event occurring after the index date. Incidence rates adjusted for demographics, specifically, age, sex, race, and ethnicity are referred to as “crude”, while incidence rates adjusted for demographics and all comorbidities are referred to as “adjusted”. Incidence rate ratios (IRR) were calculated for cases vs. controls. An α of 0.05 was considered significant.

Second, we evaluated the feasibility of associating CAA- and HTN-ICH with patient characteristics and comorbidities from the EHR using clustering analysis. Given the low event rate of ICH and limited statistical power for regression-based subtype modeling, unsupervised variable cluster analysis was used to identify patterns of comorbidities among patients with ICH (*N* = 5793). Variable clustering using the centroid-based method (SAS Varclus procedure) was selected since it groups correlated comorbidities into distinct domains based on their correlation structure and provide interpretable clusters^25^. Comorbidity status in the cluster analysis was determined one-year prior to the first occurrence of any ICH event. Cluster analysis findings were examined to assess whether real-world data patterns were consistent with the clinician-postulated ICH classifications. Statistical analyses were conducted using SAS Enterprise Guide 8.3.

## Results

### Study population

Demographic and clinical characteristics for the MCI/AD cohort (*n* = 747,475) and matched comparison cohorts are summarized in Table 1. The mean ± SD age was 77.7 ± 10.1 years and 3.6% were women. Hypertension was the most prevalent comorbidity and had a higher frequency in the MCI/AD vs. non-MCI cohort: 83.2% vs. 72.5%. The burden of other high frequency comorbidities was also consistently higher in cases vs. controls: high cholesterol (75.3% vs. 66.1%), coronary artery disease (41.2% vs. 31.6%), vasculitis (40.9% vs. 32.1%), and diabetes (37.9% vs. 29.7%). The frequency of cerebrovascular disease was more than double in the MCI/AD vs. non-MCI/AD group (22.8% vs. 9.9%).

**Table 1:** Patient characteristics.

	MCI/AD cohort(n = 747,475)	Non-MCI/AD cohort(n = 747,475)
Age, years, mean ± SD	77.7 ± 10.1	77.7 ± 10.1
Sex, n (%)		
Men	720,255 (96.4)	720,255 (96.4)
Women	27,220 (3.6)	27,220 (3.6)
Race, n (%)		
White	560,964 (75.0)	569,122 (76.1)
Black or African American	89,057 (11.9)	76,121 (10.2)
Native Hawaiian/Other Pacific Islander	6,455 (0.9)	6,210 (0.8)
American Indian/Alaskan Native	5,909 (0.8)	5,706 (0.8)
Asian	4,836 (0.6)	6,100 (0.8)
Unknown	80,254 (10.7)	84,216 (11.3)
Ethnicity, n (%)		
Non-Hispanic	652,445 (87.3)	655,575 (87.7)
Hispanic	36,877 (4.9)	30,325 (4.1)
Unknown	58,153 (7.8)	61,575 (8.2)
Comorbidities, n (%)		
Hypertension	621,668 (83.2)	541,862 (72.5)
Hypercholesterolemia	563,060 (75.3)	494,120 (66.1)
Coronary artery disease	307,640 (41.2)	236,538 (31.6)
Vasculitis	306,026 (40.9)	240,123 (32.1)
Diabetes	283,524 (37.9)	221,836 (29.7)
Dementia	260,014 (34.8)	20,166 (2.7)
Atherosclerotic coronary vascular disease	220,804 (29.5)	147,290 (19.7)
Kidney disease	197,133 (26.4)	140,227 (18.8)
Atrial fibrillation	187,763 (25.1)	143,402 (19.2)
Chronic pulmonary disease	184,987 (24.7)	136,007 (18.2)
Peripheral vascular disease	181,504 (24.3)	123,099 (16.5)
Cerebrovascular disease	170,466 (22.8)	73,923 (9.9)
Congestive heart failure	163,135 (21.8)	110,111 (14.7)
Obesity	155,245 (20.8)	129,198 (17.3)
Obstructive sleep apnea	147,561 (19.7)	100,285 (13.4)
Cancer	114,941 (15.4)	104,075 (13.9)
Post-traumatic stress disorder	99,929 (13.4)	53,422 (7.2)
Myocardial infarction	74,907 (10.0)	44,401 (5.9)
Abnormal coagulation	64,567 (8.6)	39,256 (5.3)
Seizure	39,963 (5.4)	10,261 (1.4)
Liver disease	39,513 (5.3)	24,293 (3.3)
Deep vein thrombosis	36,832 (4.9)	21,866 (2.9)
Atrial flutter	30,542 (4.1)	22,112 (3.0)
Abnormal MRI with susceptibility artifact	29,732 (4.0)	6,073 (0.8)
Percutaneous coronary Intervention/Coronary artery bypass grafting	26,235 (3.5)	20,256 (2.7)
Acute coronary syndrome/unstable angina	22,125 (3.0)	12,344 (1.7)
Hemiplegia/Paraplegia	21,256 (2.8)	6,877 (0.9)
Migraine	20,977 (2.8)	9,910 (1.3)
Rheumatic disease	20,607 (2.8)	15,349 (2.1)
Cirrhosis	17,092 (2.3)	9,957 (1.3)
Hypercoagulable state	14,255 (1.9)	8,351 (1.1)
Psoriasis	14,215 (1.9)	12,791 (1.7)
Hepatitis C	13,423 (1.8)	7,993 (1.1)
Subdural hematoma	12,975 (1.7)	3,312 (0.4)
Traumatic brain injury	12,680 (1.7)	1,498 (0.2)
Prior ICH	11,589 (1.6)	2,593 (0.3)
Peptic ulcer disease	11,343 (1.5)	6,254 (0.8)
Normal pressure hydrocephalus	9,044 (1.2)	1,103 (0.1)
Endocarditis	7,502 (1.0)	4,540 (0.6)
Ulcerative colitis	6,874 (0.9)	5,568 (0.7)
Prior SAH	6,733 (0.9)	1,435 (0.2)
Rheumatoid arthritis	6,366 (0.9)	5,129 (0.7)
Aneurysm	3,708 (0.5)	1,329 (0.2)
Crohn’s disease	3,516 (0.5)	2,990 (0.4)
Patent foramen ovale/septal defect	3,010 (0.4)	1,614 (0.2)
Human immunodeficiency virus	2,308 (0.3)	1,421 (0.2)
Sjogren	2,106 (0.3)	1,390 (0.2)
Brain tumor excluding meningioma	2,037 (0.3)	678 (0.1)
Sarcoidosis	1,970 (0.3)	1,575 (0.2)
Cerebral amyloid angiopathy	1,330 (0.2)	129 (0.02)
Arteriovenous malformation	738 (0.1)	501 (0.07)
Lupus erythematosus	689 (0.1)	476 (0.1)
Posterior reversible encephalopathy syndrome	354 (0.1)	81 (0.01)
Brain abscess	231 (0.03)	59 (0.01)
Moyamoya disease	30 (< 0.01)	6 (< 0.01)

AD, Alzheimer’s dementia; CAA, cerebral amyloid angiopathy; ICH, intracerebral hemorrhage; MCI, mild cognitive impairment; SAH, subarachnoid hemorrhage; SD, standard deviation.

Among patients in the MCI/AD cohort, 68.1% and 31.9% met the case definitions for MCI and AD, respectively. Supplemental Table 2 summarizes the demographic and clinical characteristics for MCI and AD subgroups within the MCI/AD cohort.

### ICH events

#### Crude incidence rates

The crude incidence rate (adjusted for demographics only) of any non-traumatic ICH event per 1000 person-years was 0.92 in the overall study population, 1.37 in the MCI/AD cohort, and 0.62 in the non-MCI/AD comparison group (IRR for case vs. control 2.20; *P* < 0.001). Using the clinician-postulated classification system, the event rates per 1000 person-years for CAA-related ICH, HTN-related ICH, and non-specific ICH were 0.17, 0.15, 0.59 respectively in the overall study population (Table 2A). For each of these ICH subtypes, events per 1000 person-years were higher in the MCI/AD cohort vs. the non-MCI/AD cohort: 0.27 vs. 0.11 (IRR 2.39; *P* < 0.001) for CAA-related ICH, 0.21 vs. 0.11 (IRR 1.90; *P* < 0.001) for HTN-related ICH, and 0.88 vs. 0.39 (IRR 2.24; *P* < 0.001) for non-specific ICH.

Among the MCI and AD subgroups, the crude rates of any ICH event per 1000-person years were 1.26 and 1.61, respectively. Crude event rates for CAA-, HTN-related, and non-specific ICH were 0.23, 0.20, and 0.82, respectively for MCI and 0.34, 0.24, and 1.02, respectively for AD (Supplemental Fig. 2 A).

For ICH defined by ICD codes recorded in the VAHS principal discharge diagnosis and position 1 on CMS claims, the incidence of any non-traumatic ICH was higher in MCI/AD than non-MCI/AD (0.94 vs. 0.42 per 1000 person-years) with an IRR for cases vs. control of 2.26; *P* < 0.001. The rates of each non-traumatic ICH event subtype per 1000 person-years were higher in patients with MCI/AD vs. non-MCI/AD: 0.19 vs. 0.08 for CAA-related ICH, 0.17 vs. 0.09 for HTN-related ICH, and 0.56 vs. 0.24 for non-specific ICH (Table 2A).

Among the MCI and AD subgroups, the crude rates of any ICH event per 1000-person years were 0.85 and 1.13, respectively. Crude event rates for CAA-, HTN-, and non-specific ICH were 0.16, 0.16, and 0.52, respectively for MCI and 0.26, 0.19, and 0.66, respectively for AD (Supplemental Fig. 2B).

#### Adjusted incidence rates

The adjusted rate of any non-traumatic ICH event per 1000 person-years was 0.84 in the overall study population, 1.05 in the MCI/AD cohort, and 0.68 in the non-MCI/AD comparison group (IRR for cases vs. control 1.55; *P* < 0.001). Using the clinician-postulated classification system, the adjusted event rates per 1000 person-years in the overall study population for CAA-related ICH, HTN-related ICH, and non-specific ICH were 0.15, 0.14, and 0.54, respectively (Table 2B). Events per 1000 person-years were higher in the MCI/AD cohort than in the non-MCI/AD cohort for all ICH subtypes: 0.19 vs. 0.12 for CAA-related ICH (IRR 1.63; *P* < 0.001), 0.16 vs. 0.12 for HTN-related ICH (IRR 1.35; *P*<−0.001), and 0.68 vs. 0.43 (IRR 1.59; *P* < 0.001) for non-specific ICH. Importantly, CAA-related ICH incidence in MCI/AD was 63% greater than that in controls.

Among the MCI and AD subgroups, the adjusted rates of any ICH event per 1000-person years were 1.01 and 1.17, respectively. Adjusted event rates for CAA-, HTN-, and non-specific ICH were 0.18, 0.16, and 0.66, respectively for MCI and 0.24, 0.17, and 0.73, respectively for AD (Supplemental Fig. 2 C).

For ICH defined by ICD codes recorded in the VAHS principal discharge diagnosis and position 1 on CMS claims, the incidence of any non-traumatic ICH was higher in MCI/AD than non-MCI/AD (0.71 vs. 0.46 per 1000 person-years) with an IRR for cases vs. control of 1.55; *P* < 0.001. The event rate of each non-traumatic ICH event subtype per 1000 person-years was higher in patients with MCI/AD vs. non-MCI/AD: 0.13 vs. 0.08 for CAA-related ICH (IRR 1.62; *P* < 0.001), 0.13 vs. 0.10 for HTN-related ICH (IRR 1.35; *P* < 0.001), and 0.43 vs. 0.27 for non-specific ICH (IRR 1.61; *P* < 0.001) (Table 2B).

Among the MCI and AD subgroups, the crude rates of any ICH event per 1000-person years were 0.70 and 0.77, respectively. Crude event rates for CAA-, HTN-, and non-specific ICH were 0.13, 0.13, and 0.42, respectively for MCI and 0.17, 0.13, and 0.44, respectively for AD (Supplemental Fig. 2D).

A sensitivity analysis of incidence rates was performed in which I61.0 (ICH in subcortical hemisphere of brain) was classified as CAA-related ICH, and I60.8 and I60.9 (SAH) were included as CAA-related ICH to assess the impact of incorporating SAH related diagnostic codes. We found that the incidence rates of ICH were 1.14 (crude) and 1.06 (adjusted) per 1000 person-years (Supplemental Table 4), which are similar to 0.92 (crude) and 0.84 (adjusted) per 1000 person-years among in cohorts without SAH (Table 2).

**Table 2:** ICH events per 1000 person-years A. Crude incidence rates^*^.

	Overall Study Population (N=1,494,950)	MCI/AD cohort (n=747,475)	Non-MCI/AD cohort (n=747,475)	IRR (case vs control)	P value
ICH					
Any ICH	0.92	1.37	0.62	2.2	<.0001
CAA-related ICH	0.17	0.27	0.11	2.39	<.0001
HTN-related ICH	0.15	0.21	0.11	1.9	<.0001
Non-specific ICH	0.59	0.88	0.39	2.24	<.0001
Primary ICH					
Any ICH	0.62	0.94	0.42	2.26	<.0001
CAA-related ICH	0.12	0.19	0.08	2.52	<.0001
HTN-related ICH	0.12	0.17	0.09	1.9	<.0001
Non-specific ICH	0.37	0.56	0.24	2.32	<.0001
ICH					
Any ICH	0.84	1.05	0.68	1.55	< 0.0001
CAA-related ICH	0.15	0.19	0.12	1.63	< 0.0001
HTN-related ICH	0.14	0.16	0.12	1.35	< 0.0001
Non-specific ICH	0.54	0.68	0.43	1.59	< 0.0001
Primary ICH					
Any ICH	0.57	0.71	0.46	1.55	< 0.0001
CAA-related ICH	0.11	0.13	0.08	1.62	< 0.0001
HTN-related ICH	0.11	0.13	0.1	1.35	0.0002
Non-specific ICH	0.34	0.43	0.27	1.61	< 0.0001

^*^Adjusted for Demographics Only.

^†^Adjusted for Demographics and Comorbidities.

AD, Alzheimer’s dementia; CAA-related ICH, cerebral amyloid angiopathy related intracerebral hemorrhage; HTN-related ICH, hypertension-related intracerebral hemorrhage; ICH, intracerebral hemorrhage; MCI, mild cognitive impairment.

### Cluster analysis findings

A total of 5,793 patients who had at least 1 ICH event after their study index date were included in the clustering analysis (MCI/AD, *n* = 3,741; non-MCI/AD, *n* = 2,052). Demographic and comorbidity parameters included in the cluster analysis are summarized in Supplemental Table 3.

**Fig. 1:**
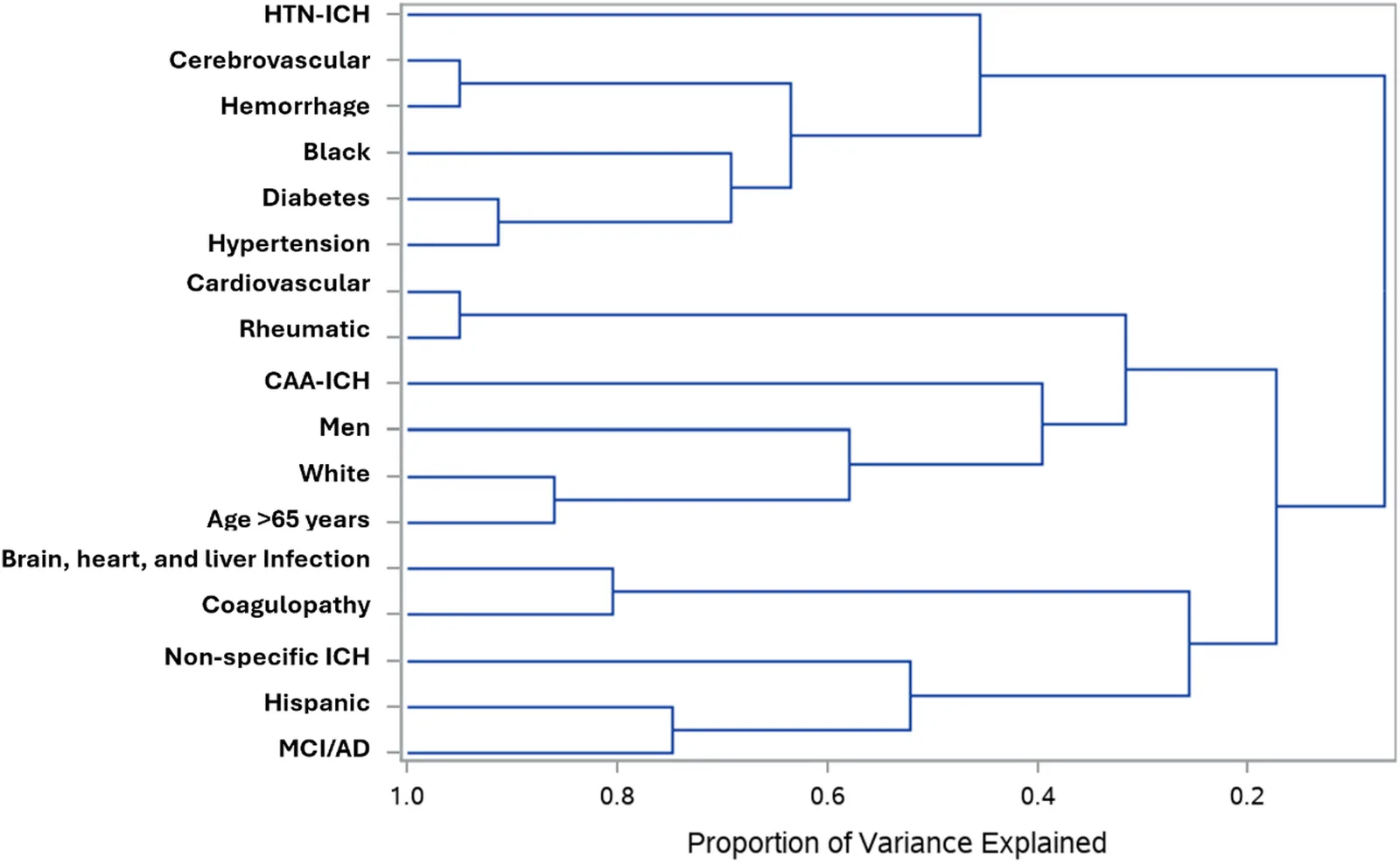
Cluster Analysis to reveal patterns of comorbidities among patients with ICH. Unsupervised variable cluster analysis was used to identify patterns of comorbidities among patients with ICH and to assess whether real-world data correlations were consistent with the clinician-postulated location-based ICH classifications. A total of 5,793 patients who had at least 1 ICH event after their study index date were included in the clustering analysis (MCI/AD, n = 3,741; non-MCI/AD, n = 2,052). AD, Alzheimer’s dementia; CAA, cerebral amyloid angiopathy; HTN, hypertension; ICH, intracerebral hemorrhage; MCI, mild cognitive impairment.

The correlation patterns of ICH types and their respective risk factors were hierarchically clustered until each attribute became a unique cluster (Fig. 1).

The first level clustering separated HTN-related ICH from the other ICH event subtypes and the second level clustering separated CAA-related ICH from non-specific ICH, hence, the cluster analysis revealed differing patterns of association among the three ICH subtypes, with their respective demographic and comorbidity characteristics mapped in the dendrogram (Fig. 1). HTN-related ICH was most closely associated with cerebrovascular disease and hemorrhage, followed by close associations with hypertension and diabetes, and further linkage to Black race. CAA-related ICH was most closely associated with White race and age > 65 years, and additional proximity to cardiovascular and rheumatic disease relative to the other ICH subtypes. Non-specific-ICH was most closely associated with Hispanic ethnicity and the MCI/AD continuum, followed by infection and coagulopathy. Thus, 3 distinctive clusters based on each respective ICH type and more closely associated health conditions explained approximately 25% of the variations in the correlation matrix.

## Discussion

This study of the VAHS/CMS administrative databases revealed that, in the overall study population of 1.5 million patients older than 50 years, the adjusted incidence of acute ICH, based on inpatient diagnostic coding for nontraumatic ICH events, was 0.84 per 1000 person-years. Patients in the MCI/AD cohort had significantly higher ICH event rates than those in the non-MCI/AD cohort for all ICH subtypes (*P* < 0.05).

The overall incidence of ICH in our study is higher than that reported in large population-based analyses such as the Framingham Heart Study (0.43 per 1000 person-years)^26^ and a large meta-analysis of 62 population-based studies (0.29 per 1000 person-years)^27^, possibly due to the risk profile unique to a Veteran population^28^. The older age (77 years) of our analysis population likely also contributed to ICH risk. Longitudinal analysis of Framingham Heart data found that in the overall population, unadjusted rate of ICH changed from 0.25 to 0.73 cases per 1000 person- years over 3 study periods; whereas, patients aged 75 and older had higher rates of 0.9 to 1.8 per 1000 person-years over the study periods^26^. ICH rates reported in our study were generally comparable to those for patients aged > 75 years in the Framingham study. Given the nature of longitudinal cohort follow-up, ICH rates in the Framingham study may not reflect risk in the general population. However, higher rates of hemorrhagic stroke, ranging from 2.7 to 5.2 per 1000 person-years have been reported in patients with AD^5^. Background rates of ICH in placebo arms of prior AD clinical trials were approximately 0.4% over 18–24 months, with reported estimates generally below 1%^29,30^.

Using the clinician-postulated, exploratory and location-based classification system for ICH ICD-10 codes in a hierarchical clustering analysis, we found that certain baseline patient characteristics had a differential association with ICH subtypes. CAA is a cerebral small vessel disease, and the association of CAA-related ICH with MCI/AD was expected^31,7^. The association of HTN-related ICH with cardiometabolic disorders, particularly hypertension and diabetes, is consistent with the known risk of nonlobar ICH with these conditions^32^. Data supporting an association with Black race are mixed^33,34^ and may warrant further study. Non-specific ICH is a broad category that includes intraventricular hemorrhage and metastatic disease, which may explain the association with older age.

### Limitations

Our ICD-10–based classification for ICH does not imply any causality in our analysis and is location-based and exploratory. Our case definition for ICH was previously validated with a PPV of 72–81%^17^, suggesting additional improvement in precision would enhance accuracy of patient identification. The anatomical location information in ICD-10 codes does not directly differentiate the underlying cause of non-traumatic ICH events, and we used a clinician-postulated classification method that relied on the coded location of the bleeding events, which is exploratory^17^. Therefore, our approach of CAA- and HTN-related ICH classification is hypothetic, and brain image analysis is needed to validate this approach in the future; even with brain image analysis, we recognize that we will not be able to account for potential variability in radiological reporting standards across hundreds of VA medical centers in the US. While CT scan might be sufficient to detect ICH that could be clinically symptomatic, specialized MRI sequences are needed to detect microhemorrhage that are often asymptomatic and without clinical manifestation. We did not perform competing risk adjustment, and our study is limited by residual confounding factors due to incomplete risk factor data (e.g., we do not have data on APOE genotype, anticoagulant use). The low number of ICH events in the study population may have hindered the ability to determine if certain patient characteristics were associated with a differential risk between CAA- vs. HTN-related ICH. Some ICH events may not have been captured due to under-coding or miscoding within the VA/CMS administrative databases and in cases where veterans received care outside of the VAHS or CMS.

A major limitation is the high proportion of non-specific ICH. Over half of ICH events were classified non-specific ICH. It is possible that some of the non-specific ICH events were either CAA- or HTN-related ICH, but this cannot be known definitively based on ICD-10 coding alone. Future studies integrating EHR imaging data are warranted to confirm validity. Another limitation is that correlations may, to some extent, reflect coding patterns in clinical practice, beyond pathophysiologic differentiation. Additionally, the MCI/AD cohort included all-cause MCI, since MCI due to AD specifically cannot be identified using ICD coding; amyloid PET, cerebrospinal fluid and plasma biomarker-based diagnosis tools can provide biological confirmation of MCI/AD status, and definitive confirmation of AD diagnosis would require postmortem pathological analysis of brain tissues.

We note that the clustering analysis is exploratory and reflects patterns of association, not causation. Additionally, given the overlapping nature of ICH pathophysiology, classifying subtypes based on the first recorded event may not capture mixed-location hemorrhages. We have re-classified HTN-related ICH based on additional ICD-10 code I61.0 to include non-traumatic ICH at subcortical hemisphere of the brain, and this increases the HTN-related ICH incidence. Therefore, our study is limited by potential misclassification, resulting in a change of CAA- and HTN-related ICH incidence. Finally, the VAHS population of mainly older men may limit generalizability of these findings. Nonetheless, our study included 27,220 female veterans with MCI/AD and matched controls, representing a large epidemiological sample.

## Conclusions

The estimated incidence of ICH events over the study period was 0.84 per 1000 person-years. The rate of CAA-related ICH in patients with MCI/AD was more than 1.6 times the rate in the non-MCI/AD comparison cohort. Our cluster analysis findings support future exploration of CAA- and HTN-related ICH events based on location-based ICD-10 coding in the combined VAHS/CMS administrative databases, with brain/neurological and cardiometabolic clusters associated with CAA- and HTN-related ICH. These findings suggest that ICD-based algorithms, when supplemented by clustering of comorbidities, may provide a venue for future empirical exploration of CAA- and HTN-related ICH in administrative data.

## Electronic Supplementary Material

Below is the link to the electronic supplementary material.

Supplementary material 1

## Data Availability

All data is maintained at the VA Healthcare System and data request can be sent to the corresponding author for VA approval.
