# Supplementary material for: Identification and characterization of intracerebral hemorrhage events in elderly veterans with alzheimer’s disease in the veterans affairs healthcare system
Source: Sci Rep. 2026 Jun 19;16:28046. doi: 10.1038/s41598-026-58486-y (PMC13550600; doi:10.1038/s41598-026-58486-y)
Supplement: Supplementary file 1 [file 41598_2026_58486_MOESM1_ESM.docx]

**Supplements**

**Figure Legend**

Supplemental Fig. 1

Proposed classification of ICH subtypes based on location information in ICD-10 codes. Inpatient nontraumatic ICH events were identified using select ICD-10 codes. ICH events were classified as related to cerebral amyloid angiopathy (CAA-related ICH) or hypertensive (HTN-related ICH) using a previously reported system (17). If ICD-10 codes did not provide adequate brain subregion/location-related information to permit classification as likely CAA-related ICH or HTN-related ICH, then events were classified as non-specific ICH.

Supplemental Fig. 2 ICH event subtypes per 1000 patient-years stratified by MCI and AD subgroups

A. The crude rates of any ICH event per 1000-person years were 1.26 and 1.61 among the MCI and AD subgroups, respectively. Crude event rates for CAA-, HTN-, and non-specific ICH were 0.23, 0.20, and 0.82, respectively for MCI and 0.34, 0.24, and 1.02, respectively for AD.

B. The crude rates of primary ICH event per 1000-person years were 0.85 and 1.13 among the MCI and AD subgroups, respectively. Crude event rates for acute CAA-, HTN-, and non-specific ICH were 0.16, 0.16, and 0.52, respectively for MCI and 0.26, 0.19, and 0.66, respectively for AD.

C. The adjusted rates of any ICH event per 1000-person years were 1.01 and 1.17 among the MCI and AD subgroups, respectively. Adjusted event rates for CAA-, HTN-, and non-specific ICH were 0.18, 0.16, and 0.66, respectively for MCI and 0.24, 0.17, and 0.73, respectively for AD.

D. The adjusted rates of primary ICH event per 1000-person years were 0.70 and 0.77 among the MCI and AD subgroups, respectively. Adjusted event rates for acute CAA-, HTN-, and non-specific ICH were 0.13, 0.13, and 0.42, respectively for MCI and 0.17, 0.13, and 0.44, respectively for AD.

**Supplemental Fig. 1.** Proposed classification of ICH subtypes based on location information in ICD-10 codes.

**ICD-10 diagnostic code for non-traumatic brain hemorrhage**

**CAA- or HTN-related ICH location-related information available in ICD-10 code?**

**Classify as non-specific ICH**

I61.2 (nontraumatic ICH in the hemisphere unspecified)

I61.5 (nontraumatic ICH that is intraventricular)

I61.8 (other nontraumatic ICH)

I61.9 (unspecified nontraumatic ICH)

**Is location-related information in ICD-10 code more consistent with etiology for CAA or HTN?**

at is unspecified)

**Classify as HTN-related ICH**

I61.0 (nontraumatic ICH in the subcortical hemisphere of the brain)

I61.3 (nontraumatic ICH in the brainstem)

I61.4 (nontraumatic ICH in the cerebellum)

**No**

**Yes**

**CAA**

**HTN**

**Classify as CAA-related ICH**

I61.1 (nontraumatic ICH in the cortical hemisphere)

I61.6 (multiple localized, nontraumatic ICH)

CAA-related ICH, cerebral amyloid angiopathy-related intracerebral hemorrhage; HTN-related ICH, hypertension-related intracerebral hemorrhage; ICH, intracerebral hemorrhage.

**Supplemental Fig. 2. ICH event subtypes per 1000 patient-years stratified by MCI and AD subgroups (A) Crude incidence rates for ICH^a^; (B) Crude incidence rates for primary ICH ^b^;(C) Adjusted incidence rates for ICH^c^; (D) Adjusted incidence rates for primary ICH.^d^**

**A.**

|  | CAA-ICH | HTN-ICH | Non-specific-ICH |
| --- | --- | --- | --- |
| MCI | 0.229828 | 0.202925 | 0.822676 |
| AD | 0.343352 | 0.236664 | 1.01568 |
| Non-MCI/AD | 0.110458 | 0.112063 | 0.392214 |

**B.**

|  | CAA-ICH | HTN-ICH | Non-specific-ICH |
| --- | --- | --- | --- |
| MCI | 0.163639 | 0.16218 | 0.51924 |
| AD | 0.260676 | 0.186775 | 0.660979 |
| Non-MCI/AD | 0.0764377 | 0.0894276 | 0.242546 |

**C.**

**D.**

|  | CAA-ICH | HTN-ICH | Non-specific-ICH |
| --- | --- | --- | --- |
| MCI | 0.17897 | 0.160292 | 0.658728 |
| AD | 0.244518 | 0.166965 | 0.733888 |
| Non-MCI/AD | 0.11505 | 0.119287 | 0.420694 |

|  | CAA-ICH | HTN-ICH | Non-specific-ICH |
| --- | --- | --- | --- |
| MCI | 0.12516 | 0.131964 | 0.423297 |
| AD | 0.165925 | 0.128506 | 0.443766 |
| Non-MCI/AD | 0.0804448 | 0.0970358 | 0.265144 |

^a^Adjusted for Demographics Only for Overall ICH

^b^Adjusted for Demographics Only for Acute ICH .

^c^Adjusted for Demographics and Comorbidities for Overall ICH

^d^Adjusted for Demographics and Comorbidities for Acute ICH.

AD, Alzheimer’s dementia; CAA-ICH, cerebral amyloid angiopathy-related intracerebral hemorrhage; HTN-ICH, hypertension-related intracerebral hemorrhage; ICH, intracerebral hemorrhage; MCI, mild cognitive impairment.

**Supplemental Table 1.** Comorbidity Diagnostic Codes.

| **Comorbidities** | **ICD-9** | **ICD-10** |
| --- | --- | --- |
|  |  |  |
| Hypertension | 401, 402, 403, 404, 405, 362.11, 437.2 | I10, I11, I12, I13, I14, I15, I16, H35.03, I67.4 |
| Hypercholesterolemia | 272.0,272.1,272.2,272.3,272.4 | E78 |
| Coronary artery disease | 410, 411, 414 | I20.0, I21, I22, I24.0, I24.1, I24.8, I24.9, I25.2, I25.1 |
| Vasculitis | 414.0, 437.4, 446.0, 447.4 | I77.6, I25, I67.7, M30.0 |
| **Diabetes** | 250.0, 250.1, 250.2, 250.3, 250.8, 250.9, | E10, E11, E13 |
| **Dementia** | 290.0, 290.1, 290.2, 290.3, 290.4, 294.0, 294.1, 294.2, 331.0, 331.1, 331.2, 331.7, 797 | F01, F02, F03, G30, G13.2, G13.8, G31.0, G31.1, G31.2, G31.83 |
| Atherosclerotic coronary vascular disease | 093.0, 410, 412, 437.3, 440, 441, 443.1, 443.2, 443.3, 443.4, 443.5, 443.6, 443.7, 443.8, 443.9, 447.1, 557.1, 557.9 | I21, I22, I25.2,  I70, I71, I73.1, I73.8, I73.9, I77.1, K55.1, K55.8, K55.9, V43.4, Z95.8, Z95.9 |
| **Cancer** | 140, 141, 142, 143, 144, 145, 146, 147, 148, 149, 150, 151, 152, 153, 154, 155, 156, 157, 158, 159, 160, 161, 162, 163, 164, 165, 166, 167, 168, 169, 170, 171, 172, 174, 175, 176, 177, 178, 179, 180, 181, 182, 183, 184, 185, 186, 187, 188, 189, 190, 191, 192, 193, 194, 200, 201, 202, 203, 204, 205, 206, 207, 208, 195.0, 195.1, 195.2, 195.3, 195.4, 195.5, 195.6, 195.7, 195.8, 199.1, 238.6 | C10, C11, C12, C13, C14, C15, C16, C17, C18, C19, C20, C21, C22, C23, C24, C25, C26, C30, C31, C32, C33, C34, C37, C38, C39, C40, C41, C43, C45, C46, C47, C48, C49, C50, C51, C52, C53, C54, C55, C56, C57, C58, C60, C61, C62, C63, C64, C65, C66, C67, C68, C69, C70, C71, C72, C73, C74, C75, C76, C81, C82, C83, C84, C85, C88, C90, C91, C92, C93, C94, C95, C96, C97, C80.1 |
| **Kidney disease** | 189.0, 198.0, 285.21, 403.01, 403.10, 403.11, 403.90, 403.91, 404.11, 404.90, 404.91, 404.93, 580.89, 580.9, 581.9, 582.89, 582.9, 583.9, 584.5, 584.8, 584.9, 585.1, 585.2, 585.3, 585.4, 585.5, 585.9, 592.0, 593.2, 593.81, 593.89, 593.9, 753.10, 753.17, 753.19, 753.3, 794.4, 996.81, V10.52, V16.51, V18.69, V45.73 | C64.1, C64.2, D63.1, E09.22, E11.22, E11.29, E13.22, I12.0, I12.9, I13.0, I13.10, I13.2, N17.0, N17.8, N17.9, N18.1, N18.2, N18.3, N18.4, N18.5, N18.9, N20.0, N20.2, N28.0, N28.1, N28.89, N28.9, N99.0, Q61.2, Q61.3, R94.4, T86.19, Z84.1, Z85.528, Z90.5 |
| Atrial fibrillation | 427.31 | I48.0, I48.1, I48.2, I48.91 |
| **Chronic pulmonary disease** | 416.8, 416.9, 506.4, 508.1, 508.8, 490, 491, 492, 493, 494, 495, 496, 497, 498, 499, 500, 501, 502, 503, 504, 505 | J40, J41, J42, J43, J44, J45, J46, J47, J60, J61, J62, J63, J64, J65, J66, J67, I27.8, I27.9, J68.4, J70.1, J70.3 |
| Peripheral vascular disease | 093.0, 437.3, 443.1, 443.2, 443.3, 443.4, 443.5, 443.6, 443.7, 443.8, 443.9, 447.1, 557.1, 557.9, 440, 441 | I70, I71, I73.1, I73.8, I73.9, I77.1, K55.1, K55.8, K55.9, V43.4, Z95.8, Z95.9 |
| **Cerebrovascular disease** | 362.34, 430, 431, 432, 433, 434, 435, 436, 437, 438 | G45, G46, I60, I61, I62, I63, I64, I65, I66, I67, I68, I69, H34.0, H34.1, H34.2 |
| **Congestive heart failure** | 398.91, 402.01, 402.11, 402.91, 404.01, 404.03, 404.11, 404.13, 404.91, 404.93, 425.4, 425.5, 425.6, 425.7, 425.8, 425.9, 428 | I43, I50, I11.0, I13.0, I13.2, I25.5, I42.0, I42.5, I42.6, I42.7, I42.8, I42.9, P29.0 |
| Obesity | 278.0, V77.8 | E66.9, Z13.89 |
| Obstructive sleep apnea | 327.23 | G47.33 |
| Post-traumatic stress disorder | 309.81 | F43.1 |
| **Myocardial infarction** | 410, 412 | I21, I22, I25.2 |
| Abnormal coagulation | 286, 287 | D65, D66, D67, D68 (except D68.[56]), D69.1, D69.3, D69.4, D69.5, D69.6 |
| Seizure | 345 | G40 |
| Liver disease | 070.22, 070.23, 070.32, 070.33, 070.44, 070.54, 070.6, 070.9, 456.0, 456.1, 456.2, 570, 571, 572.2, 572.3, 572.4, 572.5, 572.6, 572.7, 572.8, 573.3, 573.4, 573.8, 573.9 | B18, I85.0, I85.9, I86.4, I98.2, K70.0, K70.1, K70.2, K70.3, K70.9, K70.4, K71.1, K71.3, K71.4, K71.5, K71.7, K72.1, K72.9, K73, K74, K75.81, K76.5, K76.6, K76.7, K75.4, K76.0, K76.2, K76.3, K76.4, K76.8, K76.9, V42.7, Z94.4 |
| Deep vein thrombosis | - | I80, I81, I82 |
| Atrial flutter | 427.32 | I48.3, I48.4, I48.92 |
| Abnormal MRI with susceptibility artifact | 794.0 | R90, R94.0 |
| Percutaneous coronary Intervention^*^ | V45.28, V45.82 | Z98.61 |
| Acute coronary syndrome/unstable angina | 411.1,411.81,411.89 | I20.0, I24.0, I24.81, I24.89 |
| **Hemiplegia/Paraplegia** | 334.1, 344.0, 344.1, 344.2, 344.3, 344.4, 344.5, 344.6, 344.8, 344.9, 342, 343 | G81, G82, G04.1, G11.4, G80.1, G80.2, G83.0, G83.1, G83.2, G83.3, G83.4, G83.9 |
| Migraine | 346 | G43 |
| **Rheumatic disease** | 446.5, 710.0, 710.1, 710.2, 710.3, 710.4, 714.0, 714.1, 714.2, 714.8, 725 | M05, M06, M32, M33, M34, M34, M35, M31.5, M36.0 |
| Cirrhosis | 571 | K70.3, K71.7, K74, K76.1 |
| Hypercoagulable state | 289.81, 289.82 | D68.5, D68.6 |
| Psoriasis | 696 | L40 |
| Hepatitis C | 070.41, 070.51, 070.54, 070.70, 070.71 | B17.1, B18.2, B19.2, Z22.52 |
| Subdural hematoma | 432.1, 852 | I62.0, S06.5 |
| Traumatic brain injury | V15.52 | Z87.820 |
| Prior ICH | 431, 432.9 | I61, I62.9 |
| **Peptic ulcer disease** | 531, 532, 533, 534 | K25, K26, K27, K28 |
| Normal pressure hydrocephalus | 331 | G91 |
| Endocarditis | - | B33.21, I01.1, I09.1, I33, I38, I39 |
| Ulcerative colitis | 556 | K51 |
| Prior SAH | 430, 852 | I60, S06.6 |
| Rheumatoid arthritis | 714 | M05 |
| Aneurysm | 437.3 | I67.1 |
| Crohn’s disease | 555 | K50 |
| Patent foramen ovale/septal defect | 745.5 | Q21.1 |
| **Human immunodeficiency virus** | 042, 043, 044 | B20, B21, B22, B24 |
| Sjogren | 710.2 | M35.0 |
| Brain tumor excluding meningioma | 191 | C71, Z85.841 |
| Sarcoidosis | 135, 321.4 | D86 |
| Cerebral amyloid angiopathy | - | I68.0 |
| Arteriovenous malformation | 746.85 | Q24.5 |
| Lupus erythematosus | 710.0 | L93 |
| Posterior reversible encephalopathy syndrome | 348.39 | I67.83 |
| Brain abscess | 006.5, 013.1 | G06.0, A06.6, A17.81 |
| Moyamoya disease | 437.5 | I67.5 |

Comorbidities in **bold** are Charlson Comorbidity Index components.

^*^Coronary artery bypass grafting was identified using procedure codes: 0270366, 027036Z, 0270376, 027037Z, 02703D6, 02703DZ, 02703E6, 02703EZ, 02703F6, 02703FZ, 02703G6, 02703GZ, 02703Z6, 02703ZZ.

**Supplemental Table 2. Patient demographics and clinical characteristics in MCI vs AD subgroups of the MCI/AD cohort.**

|  | **MCI cohort**  **(n = 508,912)** | **AD cohort**  **(n = 238,563)** |
| --- | --- | --- |
| **Age, years, mean ± SD** | 75.5 ± 10.1 | 82.5 ± 8.4 |
| **Sex, n (%)** |  |  |
| Men | 487,503 (95.8) | 232,752 (97.6) |
| Women | 21,409 (4.2) | 5,811 (2.4) |
| **Race, n (%)** |  |  |
| White | 381,910 (75.0) | 179,054 (75.1) |
| Black or African American | 66,012 (13.0) | 23,045 (9.7) |
| Native Hawaiian/Other Pacific Islander | 4,548 (0.9) | 1,907 (0.8) |
| American Indian/Alaskan Native | 4,408 (0.9) | 1,501 (0.6) |
| Asian | 3,529 (0.7) | 1,307 (0.5) |
| Unknown | 48,505 (9.5) | 31,749 (13.3) |
| **Ethnicity, n (%)** |  |  |
| Non-Hispanic | 449,514 (88.3) | 202,931 (85.1) |
| Hispanic | 24,326 (4.8) | 12,551 (5.3) |
| Unknown | 35,072 (6.9) | 23,081 (9.7) |
| **Comorbidities, n (%)** |  |  |
| Hypertension | 416,524 (81.8) | 205,144 (86.0) |
| Hypercholesterolemia | 383,965 (75.4) | 179,095 (75.1) |
| Coronary artery disease | 196,970 (38.7) | 110,670 (46.4) |
| Vasculitis | 197,131 (38.7) | 108,895 (45.6) |
| Diabetes | 194,977 (38.3) | 88,547 (37.1) |
| Dementia | 61,731 (12.1) | 198,283 (83.1) |
| Atherosclerotic coronary vascular disease | 136,171 (26.8) | 84,633 (35.5) |
| Kidney disease | 120,881 (23.8) | 76,252 (32.0) |
| Atrial fibrillation | 115,237 (22.6) | 72,526 (30.4) |
| Chronic pulmonary disease | 123,624 (24.3) | 61,363 (25.7) |
| Peripheral vascular disease | 111,467 (21.9) | 70,037 (29.4) |
| Cerebrovascular disease | 107,461 (21.1) | 63,005 (26.4) |
| Congestive heart failure | 99,890 (19.6) | 63,245 (26.5) |
| Obesity | 121,263 (23.8) | 33,982 (14.2) |
| Obstructive sleep apnea | 118,503 (23.3) | 29,058 (12.2) |
| Cancer | 77,704 (15.3) | 37,237 (15.6) |
| Post-traumatic stress disorder | 84,706 (16.6) | 15,223 (6.4) |
| Myocardial infarction | 46,474 (9.1) | 28,433 (11.9) |
| Abnormal coagulation | 40,430 (7.9) | 24,137 (10.1) |
| Seizure | 26,347 (5.2) | 13,616 (5.7) |
| Liver disease | 30,174 (5.9) | 9,339 (3.9) |
| Deep vein thrombosis | 23,994 (4.7) | 12,838 (5.4) |
| Atrial flutter | 20,222 (4.0) | 10,320 (4.3) |
| Abnormal MRI with susceptibility artifact | 18,014 (3.5) | 11,718 (4.9) |
| Percutaneous coronary Intervention/Coronary artery bypass grafting | 17,664 (3.5) | 8,571 (3.6) |
| Acute coronary syndrome/unstable angina | 13,726 (2.7) | 8,399 (3.5) |
| Hemiplegia/Paraplegia | 14,645 (2.9) | 6,611 (2.8) |
| Migraine | 18,071 (3.6) | 2,906 (1.2) |
| Rheumatic disease | 14,295 (2.8) | 6,312 (2.7) |
| Cirrhosis | 13,258 (2.6) | 3,834 (1.6) |
| Hypercoagulable state | 10,035 (2.0) | 4,220 (1.8) |
| Psoriasis | 10,503 (2.1) | 3,712 (1.6) |
| Hepatitis C | 11,094 (2.2) | 2,329 (1.0) |
| Subdural hematoma | 7,711 (1.5) | 5,264 (2.2) |
| Traumatic brain injury | 11,752 (2.3) | 928 (0.4) |
| Prior ICH | 7,204 (1.4) | 4,385 (1.8) |
| Peptic ulcer disease | 7,121 (1.4) | 4,222 (1.8) |
| Normal pressure hydrocephalus | 5,386 (1.1) | 3,658 (1.5) |
| Endocarditis | 4,754 (0.9) | 2,748 (1.2) |
| Ulcerative colitis | 4,788 (0.9) | 2,086 (0.9) |
| Rheumatoid arthritis | 4,446 (0.9) | 1,920 (0.8) |
| Prior SAH | 4,245 (0.8) | 2,488 (1.0) |
| Aneurysm | 2,659 (0.5) | 1,049 (0.4) |
| Crohn’s disease | 2,519 (0.5) | 997 (0.4) |
| Patent foramen ovale/septal defect | 2,124 (0.4) | 886 (0.4) |
| Human immunodeficiency virus | 2,056 (0.4) | 252 (0.1) |
| Brain tumor excluding meningioma | 1,625 (0.3) | 412 (0.2) |
| Sarcoidosis | 1,633 (0.3) | 337 (0.1) |
| Sjogren | 1,610 (0.3) | 496 (0.2) |
| Cerebral amyloid angiopathy | 733 (0.1) | 597 (0.3) |
| Lupus erythematosus | 529 (0.1) | 160 (0.1) |
| Arteriovenous malformation | 470 (0.09) | 268 (0.1) |
| Posterior reversible encephalopathy syndrome | 211 (0.04) | 143 (0.1) |
| Brain abscess | 202 (0.04) | 29 (0.01) |
| Moyamoya disease | 27 (< 0.01) | 3 (< 0.01) |

**Supplemental Table 3. Parameters Included in Cluster Analysis of Patients with ICH Events (N = 5,793)**

|  | **Overall ICH** | | |
| --- | --- | --- | --- |
|  | **MCI/AD** | **Non-MCI/AD** | **All** |
|  | **n = 3,741** | **n = 2,052** | **N = 5,793** |
| **Age, mean ± SD** | 79.8 ± 8.9 | 83.0 ± 8.3 | 80.9 ± 8.8 |
| **Age > 65 years** | 3568 (95.4) | 2012 (98.1) | 5580 (96.3) |
| **Men** | 3657 (97.8) | 2012 (98.1) | 5669 (97.9) |
| **White** | 2648 (70.8) | 1632 (79.5) | 4280 (73.9) |
| **Black** | 578 (15.5) | 201 (9.6) | 779 (13.4) |
| **Hispanic** | 191 (5.1) | 79 (3.9) | 270 (4.7) |
| **Comorbidities/Comorbidity groupings** | | |  |
| **Hypertension** | 1940 (51.9) | 1235 (60.2) | 3175 (54.8) |
| **Hemorrhage*** | 441 (11.8) | 178 (8.7) | 619 (10.7) |
| **Cardiovascular†** | 2551 (68.2) | 1444 (70.4)) | 3995 (69.0) |
| **MCI/AD** | 3741 (100) | 0 (0) | 3741 (64.6) |
| **Rheumatic‡** | 1898 (50.7) | 1013 (49.4) | 2911 (50.3) |
| **Non-specific ICH** | 2179 (58.2) | 1173 (57.2) | 3352 (57.9) |
| **CAA-related ICH** | 891 (23.8) | 454 (22.1) | 1345 (23.2) |
| **Diabetes** | 816 (21.8) | 434 (21.2) | 1250 (21.6) |
| **Coagulopathy§** | 449 (12.0) | 236 (11.5) | 685 (11.8) |
| **Cerebrovascular\|\|** | 770 (20.6) | 289 (14.1) | 1059 (18.3) |
| **HTN-related ICH** | 671 (17.9) | 425 (20.7) | 1096 (18.9) |
| **Brain, heart, and liver Infection{** | 119 (2.1) | 58 (1.0) | 177 (3.1) |

**All data are presented as n (%) unless otherwise indicated.**

CAA-related ICH, cerebral amyloid angiopathy-related intracerebral hemorrhage; HTN-related ICH, hypertension-related intracerebral hemorrhage; ICH, intracerebral hemorrhage; SAH, subarachnoid hemorrhage.

^*^Hemorrhage: prior ICH, prior SAH, prior aneurysm.

^†^Cardiovascular: coronary artery disease, acute coronary syndrome, atrial flutter, atrial fibrillation, atherosclerotic cardiovascular disease, percutaneous coronary intervention, coronary artery bypass graft, patent foramen ovale.

^‡^Rheumatic: vasculitis, rheumatic disease, systemic lupus erythematosus.

**^§^**Coagulopathy: abnormal coagulation, hypercoagulable state, deep vein thrombosis.

**^||^**Cerebrovascular: Moyamoya disease, posterior reversible encephalopathy syndrome, cerebral amyloid angiopathy, arteriovenous malformation.

^{^Brain, heart, and liver infection: endocarditis, brain abscess, hepatitis C.

**Supplemental Table 4. Sensitivity Analysis of ICH Events Per 1000 Person-Years**

**A. Crude Incidence Rates^*^**

|  | **Overall Study Population (N = 1,494,950)** | **MCI/AD cohort**  **(n = 747,475)** | **Non-MCI/AD cohort (n = 747,475)** | **IRR**  **(case vs control)** | ***P*-value** |
| --- | --- | --- | --- | --- | --- |
| **Any ICH** | 1.14 | 1.70 | 0.77 | 2.22 | < 0.001 |
| **CAA-related ICH** | 0.46 | 0.69 | 0.31 | 2.24 | < 0.001 |
| **HTN-related ICH** | 0.07 | 0.1 | 0.06 | 1.82 | < 0.001 |
| **Non-specific ICH** | 0.58 | 0.87 | 0.39 | 2.26 | < 0.001 |

**B.** **Adjusted Incidence Rates^†^**

|  | **Overall Study Population (N = 1,494,950)** | **MCI/AD cohort**  **(n = 747,475)** | **Non-MCI/AD cohort (n = 747,475)** | **IRR**  **(case vs control)** | ***P*-value** |
| --- | --- | --- | --- | --- | --- |
| **Any ICH** | 1.06 | 1.32 | 0.84 | 1.57 | < 0.001 |
| **CAA-related ICH** | 0.43 | 0.54 | 0.34 | 1.58 | < 0.001 |
| **HTN-related ICH** | 0.06 | 0.07 | 0.06 | 1.23 | 0.049 |
| **Non-specific ICH** | 0.53 | 0.67 | 0.42 | 1.60 | < 0.001 |

^*^Adjusted for Demographics Only.

^†^Adjusted for Demographics and Comorbidities.

AD, Alzheimer’s dementia; CAA-related ICH, cerebral amyloid angiopathy-related intracerebral hemorrhage; HTN-related ICH, hypertension-related intracerebral hemorrhage; ICH, intracerebral hemorrhage; MCI, mild cognitive impairment.
